# Machine learning for the prediction of gram-negative bacterial secreted effectors: advances and challenges

**DOI:** 10.3389/fchem.2026.1810136

**Published:** 2026-04-21

**Authors:** Lesong Wei, Shida He, Zhengyang Fan

**Affiliations:** 1 Institute of Fundamental and Frontier Sciences, University of Electronic Science and Technology of China, Chengdu, China; 2 Yangtze Delta Region Institute (Quzhou), University of Electronic Science and Technology of China, Quzhou, China; 3 The Joint Innovation Center for Engineering in Medicine, Quzhou Affiliated Hospital of Wenzhou Medical University, Quzhou People’s Hospital, Quzhou, China; 4 Shanghai Sixth People’s Hospital Affiliated to Shanghai Jiao Tong University School of Medicine, Shanghai, China

**Keywords:** gram-negative bacterial secreted effectors, secreted effector prediction, machine learning, protein language model, negative dataset construction

## Abstract

Accurately identifying virulence-associated proteins secreted by Gram-negative pathogens is essential for elucidating bacterial pathogenic mechanisms and developing novel antimicrobial interventions. However, traditional experimental approaches for effector identification are time-consuming and labor-intensive. Recent advances in machine learning (ML), ranging from handcrafted features to context-aware embeddings derived from protein language models, have significantly improved secreted effector prediction. Here, we provide a systematic overview of ML-based methods for secreted effector prediction, surveying available database resources, negative dataset construction strategies, feature representation approaches, and model architectures spanning classical machine learning to deep learning. We discuss fundamental challenges, including data scarcity and class imbalance, evaluation bias, and model interpretability. Finally, we outline future directions encompassing multimodal data integration, meta-learning to address data limitations, and uncertainty quantification to enhance predictive robustness.

## Introduction

Gram-negative bacteria achieve pathogenicity primarily through sophisticated secretion systems that exploit their unique double-membrane envelope to translocate virulence factors into host cells (Chang et al., 2014; Costa et al., 2015; Gerlach and Hensel, 2007). Five major secretion systems, namely, type I, II, III, IV, and VI secretion systems (T1SS, T2SS, T3SS, T4SS, and T6SS), have been well-characterized as the predominant pathways in Gram-negative bacteria (Green and Mecsas, 2016). Based on translocation mechanisms, these systems are divided into pathways that are either independent of or dependent on the general secretory pathway. The former directly injects substrates into target cells, while the latter employs a two-step secretion process via the periplasm (Galán and Waksman, 2018; Hui et al., 2021; Zhao et al., 2023). These translocated substrates, termed effectors, play critical roles in bacterial pathogenicity by manipulating host cellular processes to promote survival and proliferation (Zeng and Zou, 2019). Representative pathogens such as *Salmonella* and *Legionella pneumophila* utilize T3SS and T4SS effectors, respectively, to subvert host immune defenses and establish intracellular replicative niches (Jennings et al., 2017; Ruano-Gallego et al., 2021; Zink et al., 2002). Given the key role of effectors in bacterial virulence, their accurate identification is crucial for elucidating pathogenic mechanisms and for prioritizing candidates as antibacterial targets and vaccine antigens (Qin et al., 2022).

Traditionally, the identification and validation of secreted effectors have relied heavily on wet-lab experimental approaches tailored to distinct secretion systems. For type I secreted effectors (T1SEs), hemolysis assays and C-terminal truncation mutagenesis are employed to verify effector secretion into the extracellular milieu (Alav et al., 2021; Pourhassan et al., 2023). Type II secreted effectors (T2SEs) are typically validated through biochemical fractionation and protease protection assays that confirm their transient periplasmic localization (Goll et al., 2025). For type III secreted effectors (T3SEs) and type IV secreted effectors (T4SEs), translocation into host cells is commonly demonstrated using adenylate cyclase reporter fusions or Cre recombinase-based assays (Chakravarthy et al., 2017; Guzman-Herrador et al., 2023). Type VI secreted effectors (T6SEs) are frequently characterized through interbacterial competition assays and hemolysin-coregulated protein secretion detection (Liang et al., 2015). Although these experimental techniques yield reliable results, they are inherently time-consuming, labor-intensive, and costly. Moreover, these assays are system-specific and cannot be readily scaled to accommodate the exponentially growing volume of bacterial genome sequences generated. Consequently, there is an urgent need for high-throughput computational approaches capable of identifying candidate effectors for subsequent experimental validation.

**FIGURE 1:**
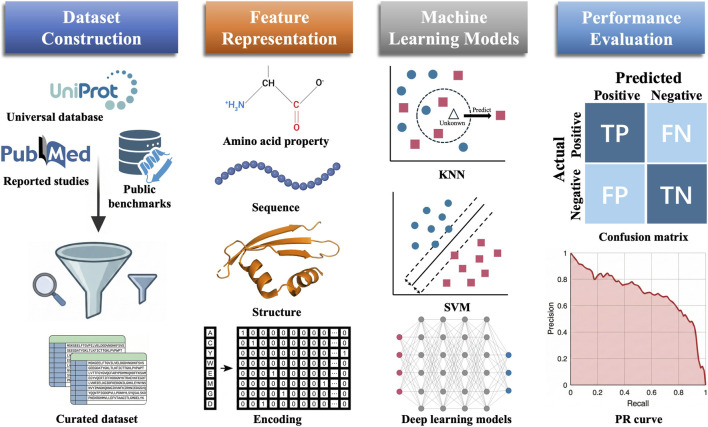
Overview of the machine learning pipeline for secreted effector identification. The workflow comprises four sequential stages from left to right: dataset construction, feature representation, machine learning modeling, and performance evaluation. In the dataset construction stage, protein sequences are collected from public databases such as UniProt and from public benchmarks reported in the literature, followed by curation and filtering. In the feature representation stage, proteins are encoded using amino acid properties, sequence-based features, structural information, or numerical encoding schemes. In the modeling stage, various algorithms are applied, such as KNN (k-nearest neighbors), SVM (support vector machine), and deep learning models. In the performance evaluation stage, prediction results are assessed using metrics such as confusion matrix entries (TP, true positive; TN, true negative; FP, false positive; FN, false negative) and precision–recall (PR) curves.

The rapid accumulation of experimentally validated effector sequences has enabled ML-based approaches to tackle this problem effectively. Rather than relying on labor-intensive biochemical assays, machine learning algorithms can identify discriminative patterns directly from amino acid sequences or structural features, enabling high-throughput screening of candidate effectors. ML-based effector prediction has advanced substantially over the past decade. Early work relied on classical models such as Support Vector Machines (SVM) and Random Forest (RF), trained on handcrafted features including Amino Acid Composition (AAC), position-specific scoring matrix (PSSM), and physicochemical descriptors (Arnold et al., 2009; Dong et al., 2013; Goldberg et al., 2016; Samudrala et al., 2009; Wang et al., 2011; Wang et al., 2013a; Yang et al., 2010; Zou et al., 2013). Although these methods achieved satisfactory performance, their dependence on predefined representations limited their ability to capture complex, context-dependent patterns in secreted effectors. With the rise of deep learning, Convolutional Neural Networks (CNNs) were introduced to detect local sequence motifs, while Recurrent Neural Networks (RNNs) captured long-range dependencies effectively (Fu and Yang, 2019; Hong et al., 2020; Hui et al., 2020; Jing et al., 2021; Li et al., 2021; Yu et al., 2021). More recently, protein language models (pLMs) such as Evolutionary Scale Modeling (ESM) (Lin et al., 2023) and ProtBERT (Elnaggar et al., 2021), pre-trained on billions of unlabeled sequences, can provide more informative features that capture global contextual dependencies (Gao et al., 2025; Li et al., 2024; Zhang Y. et al., 2023). At the same time, structure prediction, most notably AlphaFold (Jumper et al., 2021), together with rapid structural search tools like Foldseek (Van Kempen et al., 2024), now allows researchers to incorporate three-dimensional information into prediction pipelines (Peng et al., 2025). Overall, these advances have established machine learning as an efficient approach for large-scale effector discovery, significantly reducing the number of candidates requiring experimental validation. Figure 1 illustrates the general machine learning pipeline for secreted effector identification, including dataset construction, feature representation, machine learning modeling, and performance evaluation.

Here, we survey recent advances in applying machine learning to identify secreted effectors in Gram-negative bacteria. We first summarize major database resources for model training and evaluation, covering both system-specific and cross-system datasets, and then discuss negative sample construction strategies, assessing how different approaches mitigate label noise and background bias. We further review feature representations, from handcrafted descriptors to deep semantic embeddings derived from pLMs, and compare model architectures spanning classical machine learning to modern deep learning frameworks. Key limitations are also discussed, including challenges in negative dataset construction, data scarcity, evaluation bias, and model interpretability. Finally, we outline future directions encompassing multimodal data integration, meta-learning for low-data scenarios, and uncertainty quantification for robust predictions. We hope this review serves as a practical guide for developing more accurate and robust effector prediction tools.

## Database resources

The accuracy of prediction models relies heavily on the quality and coverage of training datasets. Over the past 2 decades, multiple experimentally validated resources have been curated to support ML-based effector prediction. These resources fall into two categories, namely, system-specific databases that focus on individual secretion pathways and cross-system databases that integrate data across multiple secretion systems. A summary of commonly used databases is provided in Table 1.

**TABLE 1:** Summary of curated databases.

Database	Secreted effector type	Number of effectors	URL
T3Sedb (Tay et al., 2010)	T3SE	504	http://effectors.bic.nus.edu.sg/T3SEdb
T3DB (Wang et al., 2012)	T3SE	Unclear	http://61.160.194.165:3080/T3DB/
BEAN2.0 (Dong et al., 2015)	T3SE	1,215	http://systbio.cau.edu.cn/bean
T3Enc (Hu et al., 2017)	T3SE	519	http://61.160.194.165:3080/T3Enc/index.html
SecReT4 (Bi et al., 2013)	T4SE	1884	http://db-mml.sjtu.edu.cn/SecReT4
SecReT6 v3 (Zhang et al., 2023a)	T6SE	330	https://bioinfo-mml.sjtu.edu.cn/SecReT6/
SecretEPDB (An et al., 2017)	T3SE/T4SE/T6SE	2,142	http://secretepdb.erc.monash.edu.au
BastionHub (Wang et al., 2021)	T1SE-T4SE/T6SE	2,366	http://bastionhub.erc.monash.edu

T1SE, type I secreted effector; T3SE, type III, secreted effector; T4SE, type IV, secreted effector; T6SE, type VI, secreted effector. “Unclear” indicates that the number of effectors was not explicitly reported in the original publication.

### System-specific databases

Several databases focus on individual secretion systems, providing detailed, experimentally supported annotations. For T3SS, T3SEdb (Tay et al., 2010) was the first database dedicated to T3SEs. It was constructed through systematic retrieval from PubMed literature and National Center for Biotechnology Information (NCBI) protein databases, followed by manual curation. It compiled 1,089 T3SE records from 46 bacterial species, including 504 experimentally validated effectors, 572 putative effectors, and 13 entries with unknown status. T3DB (Wang et al., 2012) took a broader approach, cataloging T3SS-related components across 26 bacterial genera and 35 representative strains, documenting not only effectors but also secretion apparatus subunits, chaperones, and regulatory factors. BEAN 2.0 (Dong et al., 2015) integrated curated literature evidence with UniProt (An et al., 2017) annotations, collecting 1,215 experimentally verified T3SEs from 221 pathogenic bacteria and providing a non-redundant benchmark set of 243 positive T3SE samples that has been widely used in comparative analyses and benchmarking studies. T3Enc (Hu et al., 2017) curated 519 experimentally validated, non-redundant T3SS effectors through systematic literature mining. Approximately 70% of these effectors were grouped into 91 homology families, while the remaining 155 were classified as singleton effectors.

For T4SS, SecReT4 (Bi et al., 2013) was constructed through genome-scale analysis of NCBI Reference Sequence data combined with manual curation of PubMed literature, cataloging 808 T4SS clusters, 10,752 core component proteins, and 1,884 effectors across 289 bacterial species. For T6SS, SecReT6 v3 (Zhang J. et al., 2023) integrates experimentally validated evidence from published studies with bacterial genome data, documenting 225 validated T6SS clusters, 330 effector proteins, and 156 cognate immunity proteins.

### Cross-system databases

While system-specific resources emphasize depth within individual secretion systems, cross-system databases provide broader coverage by aggregating effectors across multiple secretion pathways, enabling comparative analyses and large-scale data integration. SecretEPDB (An et al., 2017) curates experimentally validated effectors from T3SS, T4SS, and T6SS, comprising 2,142 proteins (1,338 T3SEs, 1,228 T4SEs, and 185 T6SEs) with explicitly traceable provenance from UniProt, NCBI Protein, and the primary literature. BastionHub (Wang et al., 2021) expands this multi-system scope to five secretion systems, integrating existing databases and curated literature to assemble 2,366 effectors (195 T1SEs, 83 T2SEs, 1,194 T3SEs, 713 T4SEs, and 181 T6SEs) across 171 bacterial species. These cross-system resources support global benchmarking and transfer-learning-based machine learning studies.

UniProt also serves as an important resource in effector prediction research. Keyword searches enable the extraction of high-confidence positive samples, while reviewed entries filtered by Gene Ontology (GO) terms and subcellular localization annotations provide reliable non-secreted proteins for constructing negative datasets (Zou et al., 2013). Together with the specialized resources described above, these curated databases provide a foundation for computational modeling of bacterial secretion system effectors and have helped standardize training and evaluation datasets across the field.

## Negative dataset construction strategies

Constructing a reliable negative dataset remains a major challenge in secreted effector prediction. “Non-effectors” represent a broad and heterogeneous category, many of which have unknown functions. Different tools use distinct strategies for negative set construction.

One common early approach samples non-effectors from bacterial proteome candidate pools (Dong et al., 2013; Goldberg et al., 2016; Wang et al., 2011; Wang et al., 2013b). For example, Dong et al. (2013) selected reviewed well-studied Gram-negative bacterial proteomes from UniProt as a candidate pool, then applied keyword-based functional annotation filtering and homology-based exclusion of known effectors to reduce noise in the negative set. While this procedure reduces the likelihood of including known or closely related effectors, the negative samples remain proxy negatives derived from proteome sampling rather than experimentally validated non-effectors. As a result, unannotated true effectors may still be included, introducing label noise that can compromise model performance in real-world applications.

When positive and negative samples come from different genomic backgrounds, models may exploit phylogenetic differences, such as GC content, rather than true secretion-related signals, resulting in inflated prediction performance (Arnold et al., 2009; Samudrala et al., 2009; Yang et al., 2010). To address this issue, some studies restrict negative samples to the same source background as positive samples. For instance, T4SEpre (Wang et al., 2014) sampled non-effectors from the same strains as the positive samples while excluding known effectors and their homologs, forcing the model to focus on intrinsic sequence patterns rather than species-specific features. However, since the negatives are still proxy negatives, label noise from unannotated effectors remains difficult to avoid.

To further reduce potential label noise in the negative set, some studies select more “conservative” background negatives, for example, reviewed intracellular or housekeeping proteins from UniProt, or non-effectors defined based on reference organisms that lack the target secretion system (Dhroso et al., 2018; Wang et al., 2019b; Zalguizuri et al., 2019; Zou et al., 2013). Such negatives typically show clear differences from effectors in secretion-related features, making them easy for models to distinguish. However, these overly simple negatives tend to overestimate model performance, since unknown effectors in real-world scenarios may not be so easily distinguished.

Another approach uses effectors from other secretion systems as negatives (Hui et al., 2020; Wagner et al., 2025; Wang et al., 2014; Xue et al., 2019; Yang et al., 2013; Yu et al., 2021). For example, when training a T4SE classifier, T1SEs, T2SEs, T3SEs, and T6SEs may serve as negative samples. This strategy ensures a “clean” negative set and avoids the risk of including unknown effectors. However, this strategy trains the model to distinguish effector subtypes rather than separate effectors from non-effectors, potentially limiting its effectiveness in whole-proteome screening.

Different negative-sampling strategies can substantially affect evaluation metrics. When negative sets are too simple, such as being dominated by intracellular proteins, models can easily achieve high metric values. In contrast, when negative samples include proteins that are sequence-similar to known effectors but functionally unrelated, model performance typically decreases, yet such settings more closely reflect real-world application scenarios. Overall, negative sample selection remains a major challenge, and how to construct negative sets that are both label-pure and sufficiently challenging remains an open question.

In practice, the choice of negative sampling strategy should be guided by the intended application. For model development and benchmarking, conservative negatives such as intracellular or housekeeping proteins can provide stable training signals and facilitate rapid performance comparison, though this may overestimate predictive performance. When the goal is to evaluate generalization under realistic conditions, proteome-derived negatives from the same genomic background as positive samples are preferable, as they better reflect the complexity of whole-proteome screening while reducing phylogenetic bias. For tasks focusing on discriminating between effector subtypes, using effectors from other secretion systems as negatives is appropriate, although this does not represent true effector versus non-effector classification. No single strategy is universally optimal, and evaluating models under diverse negative sets is recommended to provide a more comprehensive assessment of model robustness and practical utility.

## Secreted effector representation

Feature representation strongly influences model performance and generalization in secreted effector identification. Effective representations should capture discriminative information relevant to the prediction task. Current approaches to encoding protein sequences fall into several categories.

### Amino acid composition-based features

Amino acid composition-based descriptors are among the earliest and most widely used features in secreted effector identification, capturing statistical regularities within protein sequences. Basic amino acid composition (AAC) quantifies the global frequencies of twenty standard amino acids, reflecting overall compositional differences while ignoring positional information. To incorporate local sequence dependence, dipeptide composition (DPC) and tripeptide composition (TPC) are commonly used to quantify the occurrence frequencies of adjacent residue pairs and triplets, thereby capturing short-range co-occurrence patterns. Composition of k-spaced amino acid pairs (CKSAAP) enumerates residue-pair frequencies at predefined distances, preserving both pairing and relative positional information. Similarly, *k*-mer or *n*-gram features characterize sequence segment patterns at varying scales by adjusting *k*. These descriptors are computationally efficient, straightforward to implement, and relatively interpretable. However, they have limited capacity to represent physicochemical properties and long-range dependencies, and are often combined with complementary feature types to improve predictive accuracy.

### Physicochemical property–based features

Physicochemical property-based features explicitly encode biochemical attributes of amino acids, such as hydrophobicity, polarity, and charge, providing interpretable information for secreted effector characterization. Among these, the composition-transition-distribution (CTD) family is one of the most widely used. It partitions the 20 amino acids into property-based groups and quantifies the sequence from three complementary perspectives, namely, Composition, Transition, and Distribution, to characterize the organization of a given physicochemical property along the sequence. Grouped amino acid composition (GAAC) and grouped dipeptide composition (GDPC) adopt a similar grouping strategy, reducing feature dimensionality while preserving key biochemical information. Conjoint triad (CTriad) and pseudo-amino acid composition (PseAAC) further augment property-based encodings by incorporating local fragment patterns or sequence-order factors, addressing the inability of purely compositional descriptors to capture sequence dependence. These features offer computational simplicity and strong interpretability, and are frequently combined with evolutionary or other feature types to improve prediction performance.

### Evolutionary information–based features

Evolutionary information-based features capture conservation patterns and substitution preferences within homologous protein families, providing signals beyond simple compositional statistics. The foundation of this feature class is the position-specific scoring matrix (PSSM), typically constructed via iterative homology searches using PSI-BLAST (Altschul et al., 1997) to characterize evolutionary constraints and substitution tendencies at each sequence position. Numerous PSSM-derived representations have been developed to obtain fixed-length encodings and to emphasize different aspects of evolutionary signal. For example, Smoothed-PSSM (Cheng et al., 2008) applies window-based averaging to integrate signals from neighboring positions, enhancing local pattern recognition. Pse-PSSM (Chou and Shen, 2007) follows the strategy of PseAAC by transforming variable-length PSSMs into uniform-length vectors while retaining partial sequence-order information. DP-PSSM (Juan et al., 2009) explicitly models latent sequential dependencies to improve classification performance. These features effectively capture evolutionarily conserved patterns relevant to secreted effector function, improving model robustness and performance. However, they rely on database-driven homology searches, which can be computationally expensive, and they may perform poorly for proteins with few or no identifiable homologs.

### Structure features

Structural features complement sequence and evolutionary information by encoding diverse conformational and biophysical properties, such as folding states, surface accessibility, and disorder propensity. These descriptors are typically derived from structure prediction tools or statistics computed from predicted structures. Common representations include secondary structure, relative solvent accessibility, intrinsically disordered regions, distance-based residue (DR) features (Liu et al., 2017) and tertiary structure. However, structural features are constrained by substantial computational costs, particularly for tertiary structure prediction, and potential error propagation from upstream prediction tools.

### Residue-level representations

For secreted effector prediction, the primary sequence remains the most widely available source of information, and many models operate directly on residue-ordered inputs. In practice, a protein of length *L* is represented as an *n* × *L* residue-wise matrix, where each position is mapped to an n-dimensional vector before being processed by a neural network. One-hot encoding provides a straightforward discrete representation by assigning a unique binary vector to each of the 20 standard amino acids (Chen et al., 2021), but treats residues as independent symbols and encodes no explicit physicochemical information. To address this limitation, some studies employ evolutionary profiles such as PSSMs as alternative residue-level inputs, enabling models to capture functional patterns including potential enrichment of secretion-related signals in terminal regions. In deep learning settings, residue vectors can also be learned as trainable embeddings, allowing models to optimize representations for effector discrimination given sufficient training data. These sequential representations are particularly effective when paired with architectures such as CNNs or RNNs, which are well suited for capturing local motifs and long-range dependencies.

### Data-driven latent representations

Beyond handcrafted descriptors, learned latent representations have become an important alternative for protein and peptide prediction tasks. pLMs are pretrained on large-scale sequence corpora and generate context-dependent embeddings, in which each residue is represented according to its surrounding sequence context. These representations can be used either at the residue level, where position-specific embeddings are further processed by downstream architectures such as CNNs or Transformers (Yang et al., 2026; Zhang Y. et al., 2023), or at the sequence level, where residue embeddings are aggregated into a single vector for classification. Recent studies in related protein and peptide prediction tasks have shown that such pretrained representations can support diverse downstream applications (Du et al., 2024; Du et al., 2025; Kumar et al., 2025).

Compared with one-hot encodings or handcrafted statistics, pLM embeddings can capture both local motifs and long-range dependencies, making them useful for identifying remote homologs and improving generalization across species or protein families. In addition, these sequence-derived representations can be combined with predicted structural information to further enrich downstream features. For example, AlphaFold-predicted structures can be converted into structural alphabets and encoded by models such as ProstT5 (Heinzinger et al., 2024), thereby complementing sequence-based embeddings with explicit spatial context. Rather than training predictors entirely from scratch, these pretrained embeddings can be used as informative features, thereby supporting effector prediction while retaining broad biological information.

## Machine learning models for secreted effector prediction

Computational prediction of secreted effectors has progressed through three phases. Early methods relied on handcrafted features and classical machine learning classifiers. Deep learning subsequently enabled end-to-end learning from raw sequences, automatically extracting local motifs and long-range dependencies. Most recently, pLMs have provided rich and context-aware representations that further enhance predictive performance. Table 2 summarizes representative methods in this field.

**TABLE 2:** Summary of machine learning methods for secreted effector prediction.

Method (year)	Secretion system	Prediction algorithm	Feature representation
SIEVE (2009) (Samudrala et al., 2009)	T3SS	SVM	AAC, G + C content (GC), 30 N-terminal residues (SEQ), evolutionary conservation, and phylogenetic profile (PHYL)
EffectiveT3 (2009) (Arnold et al., 2009)	T3SS	Naïve Bayes (NB)	AAC, SEQ GC, and PHYL
BPBAac (2011) (Wang et al., 2011)	T3SS	SVM	N-terminal position-specific amino acid composition features
BEAN (2013) (Dong et al., 2013)	T3SS	SVM	PSSM-based k-spaced Amino Acid Pair Compositions (HH-CKSAAP)
T4EffPred (2013) (Zou et al., 2013)	T4SS	SVM	AAC, DPC, PSSM, and auto covariance transformation of PSSM
T4SEpre (2014) (Wang et al., 2014)	T4SS	SVM	Sequence-based AAC features, position-specific AAC features, secondary structure, solvent accessibility and tertiary structure
BEAN2.0 (2015) (Dong et al., 2015)	T3SS	SVM	HH-CKSAAP
pEffect (2016) (Goldberg et al., 2016)	T3SS	SVM	Sequential similarity, PSSM
Bastion6 (2018) (Wang et al., 2018)	T6SS	A two-layer SVM-based ensemble model	Sequence-based features, evolutionary information-based features, and physicochemical features
Bastion3 (2019) (Wang et al., 2019a)	T3SS	A two-layer ensemble model	Sequence-based features, evolutionary information-based features, and physicochemical features
Bastion4 (2019) (Wang et al., 2019b)	T4SS	Ensemble model	Local sequence encoding, global sequence encoding, structural descriptor encoding
DeepT3 (2019) (Xue et al., 2019)	T3SS	CNN	N-terminus one-hot embeddings
WEDeepT3 (2019) (Fu and Yang, 2019)	T3SS	CNN	k-mer word embeddings, PSSM
CNN-T4SE (2020) (Hong et al., 2020)	T4SS	CNN	PSSM, protein secondary structure and solvent accessibility and one-hot embeddings
T3SEpp (2020) (Hui et al., 2020)	T3SS	Ensemble model	Promoter information, features from signal regions, chaperone-binding domains, and effector domains
T4SE-XGB (2020) (Chen et al., 2020)	T4SS	eXtreme gradient boosting (XGBoost)	20 different types of features, including secondary structure information-based features, sequence-based features, evolutionary information-based features, global properties, terminal properties, and motifs
DeepT3 2.0 (2021) (Jing et al., 2021)	T3SS	CNN, RNN, CNN-RNN, MLP	One-hot embeddings and dictionary embeddings
DeepT3_4 (2021) (Yu et al., 2021)	T3SS/T4SS	CNN, RNN, CNN-RNN, CNN&RNN, MLP	Dictionary embeddings, AAC, DPC, PSSM
EP3 (2021) (Li et al., 2021)	T3SS	Ensemble model	PC-PseAAC, distance pair, distance-based top-n-gram, and similarity matrixes
T4SEfinder (2022) (Zhang et al., 2022)	T4SS	MLP	pLM embeddings
DeepSecE (2023) (Zhang et al., 2023b)	T1SS/T2SS/T3SS/T4SS/T6SS	CNN, Transformer	ESM embeddings
T4SEpp (2024) (Hu et al., 2024)	T4SS	Ensemble model	Multiple features
T4Seeker (2024) (Li et al., 2024)	T4SS	Long short-term memory, MLP	DR, ESM flattening features and dictionary encoding
TXSelect (2025) (Li et al., 2025)	T1SS/T2SS/T3SS/T4SS/T6SS	MLP	ESM N-terminal mean, DR, split amino acid composition general
PLM-T3SE (2025) (Gao et al., 2025)	T3SS	MLP	ProtT5 embedding and evolutionary features
CLEF (2025) (Peng et al., 2025)	T3SS/T4SS/T6SS	Contrastive learning, Transformer, MLP	pLM embeddings, evolutionary features, structural features, secretion embedding and experimental features

T1SS, type I secretion system; T2SS, type II, secretion system; T3SS, type III, secretion system; T4SS, type IV, secretion system; T6SS, type VI, secretion system; SVM, support vector machine; CNN, convolutional neural network; RNN, recurrent neural network; MLP, multilayer perceptron; XGBoost, extreme gradient boosting. AAC, amino acid composition; DPC, dipeptide composition; PSSM, position-specific scoring matrix; pLM, protein language model; ESM, evolutionary scale modeling; ProtT5, protein structure-sequence T5; DR, distance-based residue features.

### Classical machine learning approaches

Method development during this period followed two complementary trends. Feature representations expanded from terminal-segment descriptors to broader, multi-view features, while ensemble-based schemes were increasingly adopted to integrate heterogeneous predictors.

For T3SS effectors in particular, many representative methods focused on terminal signals, based on the observation that predictive information is enriched in N-terminal regions. For example, EffectiveT3 (Arnold et al., 2009) demonstrated that features derived from N-terminal peptides are “universally applicable” for predicting type III secreted proteins and implemented a machine learning predictor suitable for both individual proteins and whole proteomes. Other signal-centric approaches include Bi-profile Bayesian amino acid composition (Wang et al., 2011), which modeled position-specific residue usage patterns, and Markov model-based methods that captured adjacent-residue dependencies (e.g., T3_MM) (Wang et al., 2013a). Beyond terminal signals, subsequent studies expanded feature engineering toward multi-feature fusion, incorporating broader sequence-derived properties together with evolutionary information. SIEVE (Samudrala et al., 2009) illustrates this approach by integrating GC content, amino acid composition biases, evolutionary and phylogenetic measures, and N-terminal 30-residue features within an SVM framework.

Alongside these advances in feature engineering, ensemble learning became widely adopted across secretion systems. For T3SE prediction, Bastion3 [84] emphasized extracting features from full-length sequences and exploring multiple feature categories, then constructed a two-layer model that integrates feature groups within an ensemble-learning architecture. EP3 (Li et al., 2021) presented an ensemble predictor incorporating the Smith-Waterman algorithm and label propagation (Zhu and Ghahramani, 2002), and employed Synthetic Minority Over-sampling Technique (SMOTE) (Chawla et al., 2002) to address training-set imbalance. For T4SE prediction, T4EffPred (Zou et al., 2013) computed multiple feature families from primary sequences and trained an SVM classifier, with an additional ensemble layer designed to synthesize individual classifiers. Bastion4 (Wang et al., 2019b) systematically trained and compared multiple learners across selected features, constructing ensemble models via a majority-voting strategy. For T6SE prediction, Bastion6 (Wang et al., 2018) also extracted diverse features and developed a two-layer SVM-based ensemble model by integrating these feature groups.

### Deep learning approaches

Deep learning significantly changed how information is extracted from protein sequences. Early studies in this phase primarily employed CNNs to capture local sequence motifs and RNNs to model long-range dependencies, while exploring end-to-end strategies that integrate evolutionary and structural information with raw sequences.

DeepT3 (Xue et al., 2019) was among the first to demonstrate CNN effectiveness for T3SE identification. Using one-hot encoding of the N-terminal 100 residues as input, this method used a CNN to capture local features, demonstrating that pure sequence-derived features, without physicochemical properties or alignment-based information, can substantially improve prediction accuracy. Since one-hot encoding does not capture latent semantic relationships among residues, WEDeepT3 (Fu and Yang, 2019) introduced a more expressive representation by treating protein sequences analogously to natural language. The authors pretrained *k*-mer embeddings using Word2Vec on the UniRef50 corpus and combined these with PSSM features, enabling more effective prediction.

Extending deep learning to T4SE identification, CNN-T4SE (Hong et al., 2020) systematically compared multiple encoding schemes, including PSSM, protein secondary structure and solvent accessibility and one-hot encoding, evaluating their impact on CNN performance against T4SE datasets. Based on these benchmarks, the authors developed a voting strategy that integrates predictions from the three best-performing models, achieving reduced false-positive rates while maintaining sensitivity.

Beyond encoding and single-model optimization, subsequent efforts focused on architectural diversity and multi-model integration. DeepT3 2.0 (Jing et al., 2021) benchmarked CNN, RNN, and CNN-RNN hybrid architectures across different sequence-length settings, demonstrating that a voting-based meta-predictor integrating multiple models improves accuracy and coverage for genome-scale scanning. T3SEpp (Hui et al., 2020) implemented a more comprehensive pipeline combining homology-based screening modules with deep learning predictors (T3SEdnn and T3SErnn) and subcellular localization tools, deriving final probabilities from weighted integration of all module outputs, a strategy that effectively mitigates the high false-positive rates inherent in single-model approaches.

Conventional sequence representations, including manually curated physicochemical features, multiple sequence alignment (MSA)-derived evolutionary profiles such as PSSM, and one-hot encoding, struggle with orphan proteins lacking detectable homologs and remain sensitive to alignment quality. In contrast, pLMs can learn richer contextual representations directly from raw sequences through large-scale pretraining, without relying on alignment-based methods.

For T4SE prediction, T4SEfinder (Zhang et al., 2022) encoded sequences with pLM embeddings and classified them via multilayer perceptrons (MLPs) or bidirectional long short-term memory networks (BiLSTM). The authors demonstrated that pLM-derived representations paired with simple classifiers markedly outperform PSSM-based approaches without requiring MSAs, while enabling genome-scale screening at substantially reduced computational cost. T4Seeker (Li et al., 2024) pursued improved robustness through multi-level feature fusion, integrating amino acid composition, distance-based residue (DR) descriptors, ESM embeddings, and BiLSTM-derived features for MLP-based classification. T3SEs and T6SEs were incorporated as hard negative samples during training, forcing the model to capture T4SE-specific signatures rather than generic secretion motifs. To mitigate the high false-positive rates associated with single-strategy approaches, T4SEpp (Hu et al., 2024) integrated homology-search units, traditional ML modules, and attention-based transfer-learning models built on pLM embeddings, with final predictions derived from weighted aggregation of module outputs.

Beyond binary classifiers targeting individual secretion systems, several unified frameworks have been developed for multi-class effector prediction. DeepSecE (Zhang Y. et al., 2023) addresses six-class classification of secreted effectors by feeding pLM embeddings through convolutional layers for local motif detection, followed by Transformer encoders to capture long-range dependencies. TXSelect (Li et al., 2025) adopts a multi-task learning framework to jointly identify Type I, II, III, IV, and VI effectors using a shared backbone with task-specific heads, integrating ESM-derived features with conventional descriptors. This shared-backbone design addresses generalization limitations of single-task models. To better integrate pLM representations with heterogeneous biological features, CLEF (Peng et al., 2025) employs a contrastive learning framework with a dual-encoder architecture. One encoder transforms frozen pLM representations, while the other projects modality features into a shared latent space. Using Information Noise Contrastive Estimation loss, CLEF learns cross-modality representations by aligning paired inputs during pretraining, achieving high sensitivity for intestinal pathogen effectors while enabling downstream inference directly from protein sequences.

### Comparative analysis across methodological approaches

While methodological advances have markedly improved effector prediction, each approach has its own strengths and limitations. Classical ML approaches rely on handcrafted sequence features that have limited capacity to capture complex, nonlinear sequence relationships. Moreover, commonly used descriptors such as PSSM-derived evolutionary profiles depend on MSA quality and the availability of homologous sequences, limiting their effectiveness for orphan proteins. Deep learning models address this limitation by learning hierarchical representations directly from raw sequences, enabling automatic discovery of motifs and long-range patterns. pLM-based methods further improve sequence representation by generating residue embeddings from single sequences, providing more informative features without relying on MSAs.

The three approaches also differ in interpretability. Classical approaches offer greater interpretability, as handcrafted features can often be directly linked to biological hypotheses. Deep learning and pLM-based models are less interpretable. Although *post hoc* techniques such as attention visualization and gradient-based attribution can highlight informative sequence regions, these signals rarely provide clear mechanistic insights.

Despite their higher predictive accuracy, deep learning and pLM-based methods come with practical trade-offs. Deep neural networks require substantially greater computational resources and memory compared with classical models such as SVMs. This is especially pronounced for pLMs, which often contain hundreds of millions to billions of parameters. Additionally, many effector prediction datasets remain relatively small, increasing the risk of overfitting for large neural architectures. In contrast, traditional machine learning models trained on carefully designed sequence features are typically more computationally efficient and can remain competitive in data-limited settings. Therefore, selecting an appropriate method requires balancing predictive performance, computational cost, and data availability.

Beyond architectural differences, existing methods also vary substantially in dataset construction, negative sampling strategies, and evaluation protocols. As a result, reported performance values are often not directly comparable across studies. Many early studies rely on curated or simplified negative sets, which may overestimate predictive performance, whereas more recent approaches attempt to incorporate harder negatives or cross-system settings to better reflect real-world scenarios. In addition, evaluation schemes differ across studies, further complicating direct comparison. These inconsistencies highlight the need for standardized benchmark datasets and unified evaluation protocols to enable fairer and more meaningful comparison across methods.

## Performance evaluation

Evaluating model performance in secreted effector prediction requires multiple complementary metrics, as no single measure can fully capture classification quality. Accuracy (ACC) provides an overall measure of correct predictions, while Sensitivity (SN) and Specificity (SP) quantify the model’s ability to correctly identify true positives and true negatives, respectively. Given the inherent class imbalance commonly observed in biological datasets, where non-effectors typically far outnumber effectors, the Matthews Correlation Coefficient (MCC) offers a more reliable and unbiased assessment of classification performance across skewed distributions. The MCC ranges from −1 to 1, where 1 indicates perfect prediction, 0 indicates performance no better than random guessing, and −1 indicates complete disagreement between predictions and true labels. Unlike ACC, which can appear misleadingly high when a model predominantly predicts the majority class, MCC remains informative under class imbalance because it better reflects the balance among all four outcomes in the confusion matrix. In practice, higher positive MCC values indicate better classification performance.

Additionally, the F1 score, defined as the harmonic mean of Precision (PR) and Sensitivity, effectively balances the trade-off between false positives and false negatives, making it particularly informative for imbalanced classification tasks. These metrics are calculated as follows:

$$\begin{array}{c} \text{ACC}=\frac{\text{TP}+\text{TN}}{\text{TP}+\text{FP}+\text{TN}+\text{FN}},\\ \text{SN}=\frac{\text{TP}}{\text{TP}+\text{FN}},\\ \text{SP}=\frac{\text{TN}}{\text{TN}+\text{FP}},\\ \mathrm{PR}=\frac{\text{TP}}{\text{TP}+\text{FP}},\\ F1=\frac{2}{1/\text{SN}+1/\mathrm{PR}},\\ \text{MCC}=\frac{\left(\text{TP}\times \text{TN}\right)-\left(\text{FN}\times \text{FP}\right)}{\sqrt{\left(\text{TP}+\text{FN}\right)\times \left(\text{TP}+\text{FP}\right)\times \left(\text{TN}+\text{FP}\right)\times \left(\text{TN}+\text{FN}\right)}}, \end{array}$$

where TP and TN represent correctly classified effectors and non-effectors, while FP and FN denote misclassified non-effectors and effectors, respectively. Beyond these threshold-specific metrics, the Receiver Operating Characteristic (ROC) curve plots the true positive rate against the false positive rate across all thresholds. However, ROC-based metrics can appear falsely optimistic on highly imbalanced datasets, where large numbers of true negatives dominate the evaluation. In genome-scale effector screening, non-effectors typically greatly exceed true effectors. Precision–Recall (PR) curves are more informative in this setting, as they focus on positive-class performance. Therefore, PR-based metrics together with careful threshold calibration may provide a more realistic assessment of model utility in practical screening scenarios.

## Current limitations

Despite significant methodological advances, several fundamental challenges still limit the development and reliable deployment of secreted effector predictors. These limitations span negative dataset construction, data availability, model evaluation, and biological interpretability.

### Negative dataset construction

Constructing reliable negative datasets remains a major unresolved problem in secreted effector prediction. Because non-effectors represent a broad and heterogeneous category, no existing sampling strategy can simultaneously guarantee label purity and realistic task difficulty. Proteome-derived negatives may contain unannotated effectors, whereas conservative negatives such as housekeeping proteins often make classification artificially easy. Using effectors from other secretion systems avoids label contamination but shifts the task toward subtype discrimination rather than distinguishing effectors from non-effectors. As a result, how to construct reliable and realistic negative datasets remains an unresolved challenge in both model training and performance evaluation.

### Limited data availability and class imbalance

The scarcity of experimentally validated effectors, together with severe class imbalance, remains a major obstacle to developing high-performance prediction models. In addition, data availability varies considerably across secretion systems. Compared with the relatively abundant datasets for T3SEs and T4SEs, experimentally validated T1SEs and T2SEs remain far fewer in number, further aggravating the imbalance problem. For example, in DeepSecE, T3SEs and T4SEs numbered 406 and 504, respectively, whereas only 128 T1SEs and 68 T2SEs were available. Such imbalance can bias decision boundaries toward majority classes and increase the risk of overfitting, particularly in deep learning models trained on limited samples. Under these conditions, models may capture phylogenetic noise rather than broadly generalizable secretion signals. Although various resampling strategies have been used to alleviate class imbalance, their suitability for highly heterogeneous effector datasets remains uncertain, especially in deep representation learning settings where simple synthetic augmentation may not preserve biologically meaningful variation.

### Evaluation bias from inadequate train-test separation

Even with carefully curated datasets, redundancy reduction using tools such as CD-HIT clustering at 60%–90% sequence identity has become standard practice for removing near-duplicate sequences. However, such filtering mainly eliminates highly similar sequences and does not prevent homologous proteins from the same families from appearing in both training and test sets. In such cases, models may achieve artificially high accuracy by recognizing homologous patterns rather than learning generalizable secretion signals.

Another potential source of evaluation bias arises when embeddings derived from pLMs are used as input features. Models such as ESM are pre-trained on hundreds of millions of sequences from databases like UniProt, meaning some sequences in downstream datasets may have appeared during pretraining. While this does not constitute label leakage, the model may have already learned representations for the same or closely related sequences, potentially inflating performance on familiar inputs.

To mitigate these biases, future studies should consider adopting stricter clustering thresholds and cluster-aware evaluation strategies. Cold-start splitting, where entire sequence clusters are assigned exclusively to either training or test sets, provides a more rigorous approach to ensuring test-set independence and obtaining realistic estimates of model generalization.

### Model interpretability and biological validation

Beyond data and evaluation concerns, interpretability remains a challenge. Traditional linear or tree-based models offer intuitive interpretability through feature weights or Gini importance (Guyon and Elisseeff, 2003), facilitating identification of secretion signals. Deep learning models achieve better performance but are much harder to interpret, often functioning as black boxes. While *post hoc* methods (Wong et al., 2024; Yuan et al., 2022) can highlight important features, the computationally identified “importance” often fails to align with actual biochemical mechanisms. A major gap is the lack of experimental validation for computationally inferred features. Future studies would benefit from closer collaboration between computational and experimental researchers, employing site-directed mutagenesis or structural approaches to verify whether model-identified residues mediate interactions between effectors and secretion machinery.

## Outlook

ML has been widely applied to address key challenges in secreted effector identification and characterization. Numerous candidate effectors discovered using ML models have been experimentally validated for their translocation and biological functions, demonstrating the value of ML in effector discovery. ML will likely continue to accelerate research in this field, helping scientists elucidate pathogenic mechanisms and prioritize candidates for antimicrobial therapies and vaccine development. Based on the limitations discussed above, we outline three directions for future work: (i) multimodal dataset construction, (ii) meta-learning to address data scarcity and class imbalance, and (iii) generalizability assessment and uncertainty quantification.

### Multimodal dataset construction

With breakthroughs in structure prediction methods such as AlphaFold, structural information has become increasingly accessible for large-scale computational analysis. Numerous studies have shown that incorporating structural features can improve downstream predictive performance compared with sequence-only representations. Beyond sequence and structure, future datasets should integrate diverse effector-related knowledge, including protein functional annotations, GO terms, and binding site information. Incorporating such expert-curated knowledge may enable more comprehensive and biologically meaningful representations.

However, integrating heterogeneous data sources introduces technical challenges. In particular, modality collapse can occur when highly discriminative features from one source dominate the optimization process, causing the model to underutilize signals from other modalities. Addressing this issue may require carefully designed fusion strategies, such as feature alignment, cross-modal attention mechanisms, or gradient balancing techniques, to better integrate complementary information across modalities.

Furthermore, constructing unified benchmark datasets encompassing multiple secretion systems with standardized annotations would enable fair model comparison and accelerate methodological advances. Such multimodal benchmarks could also support multi-task learning frameworks, where shared representations across related prediction tasks enhance performance on individual classification problems.

### Meta-learning to address data scarcity and class imbalance

Complementing improved datasets, meta-learning offers a promising framework for addressing the scarcity of experimentally validated effectors and extreme class imbalance across secretion systems, with orders-of-magnitude differences between well-studied systems such as T3SS and T4SS and underrepresented ones such as T1SS and T2SS. Meta-learning can enhance generalization for rare classes by capturing shared patterns across tasks or protein families, achieving robust performance even with limited training samples. By learning to generalize across functional categories, models can transfer knowledge from data-rich systems to identify effectors in the long-tail distribution. Inspired by successful applications such as Mutual Information Maximization Meta-Learning for peptide bioactivity prediction (He et al., 2022) and ActFound for bioactivity analysis (Feng et al., 2024), future research should explore few-shot or zero-shot learning architectures for effector prediction, which are essential for discovering novel effector families lacking annotated homologs. Furthermore, integrating meta-learning with multimodal data, including sequences, predicted structures, and protein annotations, would enable models to dynamically adapt to newly sequenced strains with minimal fine-tuning, accelerating the discovery of novel effectors.

### Generalizability assessment and uncertainty quantification

Finally, reliable effector predictors require more than strong benchmark performance; they must also generalize well to new genomes and clearly indicate their confidence in each prediction. Current evaluation practices, typically based on random cross-validation after redundancy reduction, often overestimate real-world performance, as noted above. Systematic generalizability assessment should become standard practice, including cold-start evaluation against phylogenetically distant organisms, held-out testing on underrepresented secretion systems, and explicit reporting of performance stratified by sequence similarity to training data. These analyses would help define the conditions under which a predictor remains reliable.

Uncertainty quantification is equally important. Conventional effector classifiers typically output a single probability score without quantifying prediction reliability. Future architectures should incorporate uncertainty quantification techniques to provide confidence intervals or reliability metrics for each prediction. These confidence estimates can effectively identify low-confidence predictions arising from insufficient training examples, helping experimentalists prioritize candidates for downstream validation.

## Conclusion

The prediction of secreted effector proteins in Gram-negative bacteria has advanced significantly over the past decade, driven by the integration of diverse feature representations and increasingly sophisticated machine learning architectures. As summarized in this review, the field has progressed from early classifiers trained on simple amino acid composition to modern approaches leveraging deep semantic embeddings from pLMs, with each generation of methods offering improved predictive accuracy and broader applicability across secretion systems.

Despite these advances, several fundamental challenges remain. Data scarcity and extreme class imbalance continue to limit model training, particularly for effectors from understudied systems such as T1SS and T2SS. The construction of reliable negative samples remains a critical yet often overlooked issue, as inappropriate sampling strategies can introduce substantial bias and inflate performance estimates. Furthermore, most current models function as black boxes, offering limited insight into the biological features driving their predictions, which remains a significant barrier to both mechanistic understanding and broader adoption by experimentalists.

Several technical developments are likely to drive future progress. The integration of multimodal features, combining sequence, structure, and genomic context, may enable biologically richer representations. Meta-learning and few-shot learning strategies could address data limitations for understudied systems. Advances in uncertainty quantification will enable models to flag low-confidence predictions, guiding more efficient experimental validation.

In conclusion, machine learning has become a powerful tool for large-scale effector discovery. By addressing current limitations and embracing emerging methodologies, researchers will be able to develop more accurate and robust prediction tools.
